# Factors Impacting Microwave Ablation Zone Sizes: A Retrospective Analysis

**DOI:** 10.3390/cancers16071279

**Published:** 2024-03-26

**Authors:** René Michael Mathy, Athanasios Giannakis, Mareike Franke, Alain Winiger, Hans-Ulrich Kauczor, De-Hua Chang

**Affiliations:** 1 Department of Diagnostic and Interventional Radiology, University Hospital of Heidelberg, 69120 Heidelberg, Germany; athgiannakis@uoa.gr (A.G.);; 2 2nd Department of Radiology, University General Hospital, “ATTIKON” Medical School, National and Kapodistrian University of Athens, 12462 Athens, Greece; 3 Department of Radiology, Cantonal Hospital of Lucerne, 6000 Lucerne, Switzerland

**Keywords:** microwave ablation, thermal ablation, hepatocellular carcinoma, liver metastasis, liver cirrhosis, ablation zone

## Abstract

**Simple Summary:**

Ablation zone volumes (AZV) following microwave ablation (MWA) for liver tumours exhibit considerable variability, posing challenges for precise therapy planning. In this retrospective analysis, we aimed to identify factors influencing AZV using a specific device designed to restrict the ablation zone past the needle tip. Furthermore, we assessed the efficacy of a ‘surgical mode’ in expanding the device’s application range compared with the conventional ‘standard mode’. Notably, the observed AZVs were smaller than the manufacturer’s predictions. We found significantly larger AZVs in non-perivascular tumour locations and in hepatocellular carcinoma, compared with metastases. Despite these variations, the use of the ‘surgical mode’ did not yield significantly larger AZVs compared with the ‘standard mode’. These insights contribute to understanding the nuances of AZV and optimising the application of MWA for liver tumours.

**Abstract:**

Purpose: Evaluation of the influence of intrinsic and extrinsic conditions on ablation zone volumes (AZV) after microwave ablation (MWA). Methods: Retrospective analysis of 38 MWAs of therapy-naïve liver tumours performed with the NeuWave PR probe. Ablations were performed either in the ‘standard mode’ (65 W, 10 min) or in the ‘surgical mode’ (95 W, 1 min, then 65 W, 10 min). AZV measurements were obtained from contrast-enhanced computed tomography immediately post-ablation. Results: AZVs in the ‘standard mode’ were smaller than predicted by the manufacturer (length 3.6 ± 0.6 cm, 23% below 4.7 cm; width 2.7 ± 0.6, 23% below 3.5 cm). Ablation zone past the tip was limited to 6 mm in 28/32 ablations. Differences in AZV between the ‘surgical mode’ and ‘standard mode’ were not significant (15.6 ± 7.8 mL vs. 13.9 ± 8.8 mL, *p* = 0.6). AZVs were significantly larger in case of hepatocellular carcinomas (HCCs) (*n* = 19) compared to metastasis (*n* = 19; 17.8 ± 9.9 mL vs. 10.1 ± 5.1 mL, *p* = 0.01) and in non-perivascular tumour location (*n* = 14) compared to perivascular location (*n* = 24, 18.7 ± 10.4 mL vs. 11.7 ± 6.1 mL, *p* = 0.012), with both factors remaining significant in two-way analysis of variance (HCC vs. metastasis: *p* = 0.02; perivascular vs. non-perivascular tumour location: *p* = 0.044). Conclusion: Larger AZVs can be expected in cases of HCCs compared with metastases and in non-perivascular locations. Using the ‘surgical mode’ does not increase AZV significantly.

## 1. Introduction

For the treatment of hepatocellular carcinomas (HCCs) and liver metastases, thermal ablative procedures have been firmly established and routinely employed in our department. Microwave ablation (MWA) is based on thermal heating of the target volume to induce targeted tumour necrosis [1]. MWA induces dielectric hysteresis of polar molecules (primarily water) due to an oscillating electric field; therefore, it does not rely on electrical conductivity, leading to a continuous energy input during the treatment period [2] and in a higher thermal efficiency compared with radiofrequency ablation or cryoablation [3]. A distinction can be established between an inner zone of direct heating and an outer zone of heating via thermal conductivity, which is more sensitive to external influences [2].

In terms of the AZV size, achieving optimal predictability is paramount to ensure a thorough ablation and to avoid rest tumours or local recurrence while simultaneously minimising the risk of complications, such as vessel occlusion leading to parenchymal ischemia. However, manufacturers’ data mostly depend on ex vivo data. Clinical data demonstrate a deviation, usually smaller, from these values as well as a significant variance in the AZVs achieved [4,5,6]. Therefore, a reliable prediction of the AZV is still difficult [7,8]. Potential influencing factors include the proximity of adjacent large vessels [9], tissue perfusion [10,11] including blood velocity and blood flow direction towards the needle [12], tumour type, and surrounding liver tissue characteristics (healthy parenchyma versus cirrhotic or steatotic conditions) [10,13,14]. Moreover, in vivo studies, notably those by Hines-Peralta et al. [15] and Bedoya et al. [16], reveal a non-linear relationship between AZV and ablation time. The primary reason for this is that AZV seems to approach a maximum with longer ablation durations [15,17]. Thus, summarising various ablation parameters under an ablation volume/energy ratio may be imprecise.

NeuWave (NeuWave MWA, Ethicon, Madison, WI, USA) offers two needles for liver ablation: the LK needle, with a high achievable power (up to 140 watts), and the PR needle, where the maximum achievable power is significantly lower (up to 65 watts). The system works at a frequency of 2.45 GHz, which is similar to most other MWA systems on the market. In contrast to other MWA systems which use fluid cooling, the NeuWave system uses a CO_2_ cooling system. According to the manufacturer’s instructions, which are based on ablations in ex vivo bovine liver, an AZ of up to 6.0 cm × 3.9 cm can be attained with the LK-XT needle (AZV: 47.8 mL), while an AZ of 4.7 cm × 3.5 cm can be achieved with the PR-XT needle (AZV: 30.1 mL). However, the PR needle limits the transfer of energy beyond its tip. This attribute proves beneficial in safeguarding vulnerable structures such as blood vessels, bile ducts, and extrahepatic organs during the ablation process. When using the PR needle, ablation can be performed in a ‘surgical mode’, where 95 W can be employed for a maximum of 1 min followed by an ablation with 65 W for 10 min. Subsequently, this ‘surgical mode’ has the potential to narrow the gap between PR and LK needles by extending the application range of the PR needle towards ablating larger liver lesions while maintaining the often-crucial safety feature of confining heat proximal to the needle tip.

This study aims to achieve two primary goals: to document the range of AZVs in vivo using the PR needle and to identify potential influencing factors associated with this variance. Additionally, we will investigate whether the ‘surgical mode’ actually leads to larger AZVs than the ‘standard mode’.

## 2. Materials and Methods

For this single-centre retrospective analysis, microwave ablations of HCCs or liver metastases performed with the NeuWave PR probe (NeuWave MWA, Ethicon, Madison, WI, USA) between September 2020 and February 2023 were included. Only ablations performed either with 65 W for 10 min, which is the highest achievable energy output in the ‘standard mode’, or in the ‘surgical mode’ with 95 W for 1 min followed by 65 W for 10 min were included. Exclusion criteria included local therapy (TACE or ablation) of the ablated lesion prior to treatment, no available contrast-enhanced computed tomography (CT) immediately after ablation, lack of measurability of the AZ due to overlap with previous AZs, artefacts, post-ablation in the same session, or tract ablation before performing the contrast-enhanced series.

All ablations were conducted using a NeuWave^TM^ PR-XT needle. Ablation was performed under general anaesthesia. Needle placement was performed under CT guidance (Siemens Somatom Edge, Siemens, Erlangen, Germany). After placement, ablation was performed either in the ‘standard mode’ with 65 W for 10 min or in ‘surgical mode’ with 95 W for 1 min followed by 65 W for 10 min. Subsequently, a contrast-enhanced control scan was performed, typically with the needle still in place. This evaluation commonly included the venous contrast phase and, in most instances, the arterial phase to evaluate the success of the ablation; 1 mm layers with soft tissue kernels were reconstructed. If incomplete ablation was assumed in this scan, re-ablation was performed immediately thereafter.

**Figure 1:**
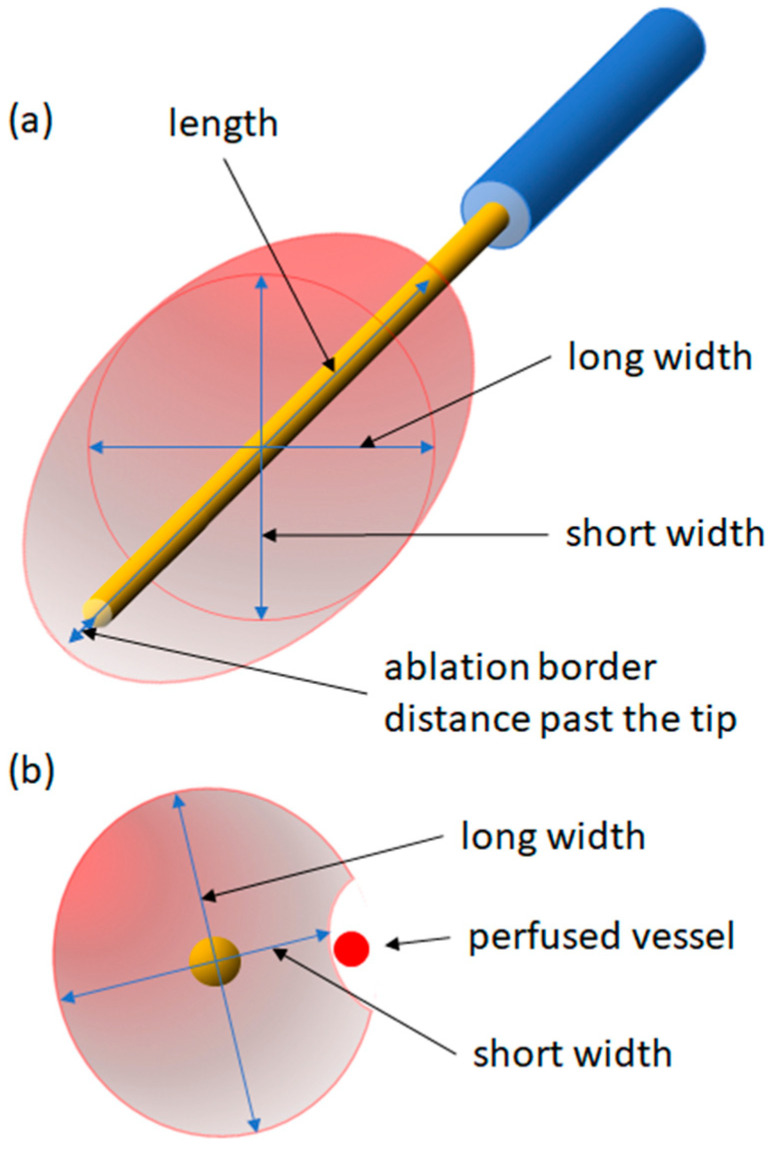
Measurements of AZ in three planes. (**a**) 3-dimensional view. (**b**) 2-dimensional view perpendicular to the probe.

AZ sizes were measured in the contrast scan immediately after ablation in multiplanar reconstructions in three planes (parallel and orthogonal to the needle, Figure 1a). The AZV was approximately calculated as an ellipsoid as follows: AZV = 1/6 × π × length × long width × short width. The sphericity index (S) was defined as the volume of the ablation divided by the volume of an ideal sphere using the AZ length [5,15]. Thus, the resulting formula is as follows:

(1) $$S=\frac{\frac{1}{6}\times \pi \times length\times long\;width\times short\;width}{\frac{1}{6}\times \pi \times {length}^{3}}=\frac{\mathrm{long width}\times \mathrm{short width}}{{\mathrm{length}}^{2}}$$

If the needle was still in the ablation position during the control scan, the AZ boundary distal to the needle tip was measured.

If a perfused vessel was present within the AZ after ablation or if a vessel caused a significant ‘dent’ in the AZ, this was interpreted as the presence of a heat-sink effect (Figure 1b).

The size of the ablated lesion was measured in the axial plane in the long and short axes on the last magnetic resonance imaging (MRI) or CT performed before the examination. Lesions were defined as subcapsular if the distance to the liver capsule was <5 mm. A lesion was perivascular if it bordered a vessel with a diameter >3 mm within a distance of <5 mm. Moreover; the lesion vascularity (hypo- or hypervascularised) was recorded.

Follow-ups were mostly conducted using MRI and alternatively by CT. Residual tumours were defined as remaining tumour manifestations at the AZ margin that were detectable during the first follow-up. Local recurrences were tumour manifestations directly at the AZ margin that were detected in one of the subsequent follow-up controls. Intrahepatic tumour recurrences were defined as new HCC-susceptible or metastasis-susceptible lesions occurring during follow-ups that were absent prior to ablation. Extrahepatic metastasis was defined as newly detected distant metastases during follow-up examination.

Patient-based variables such as age, sex, Child–Pugh classification, number of tumours, presence of cirrhosis, and Child score were recorded.

Descriptive statistics were calculated for collected data, with means and standard deviations determined for normal distributed data and median and interquartile range for non-normal distributed data. Yate’s corrected chi-square test was used to compare categorical data. Continuous data were compared using Student’s *t*-test or two-way analysis of variance (ANOVA). The Pearson correlation coefficient was calculated to measure correlations between two sets of continuous data. Statistical significance was set at *p* < 0.05. Statistical analysis was performed using SPSS version 27 (IBM Corporation, Armonk, NY, USA).

## 3. Results

The study included 38 microwave ablations conducted in 37 patients, with 19 ablations dedicated to HCC and another 19 ablations targeting liver metastases. The mean tumour size was higher for HCC (1.72 ± 0.39 cm vs. 1.21 ± 0.50 cm, *p* = 0.001). Naturally, some differences were observed between the two groups in baseline characteristics (Table 1): Liver cirrhosis was present in all HCC cases but in only one of the metastatic cases. Correspondingly, portal vein hypertension was present in 12/19 HCC cases but not in any of the metastatic cases. Two HCC cases had Child–Pugh B cirrhosis, and the others were Child–Pugh A. Hypervascularity was present in all HCCs but in only 6/19 metastatic cases. Systemic therapy had been previously conducted in 17/19 metastatic cases but in only 1/19 HCC cases. Pericapsular tumour location (distance to capsule ≤ 5 mm) was present in 11/19 HCC cases but in only 5/19 metastatic cases. A perivascular tumour location (distance ≤ 5 mm to a vessel with a diameter ≥ 3 mm) was present in 14/19 metastasis cases and in 10/19 HCC cases.

**Figure 2:**
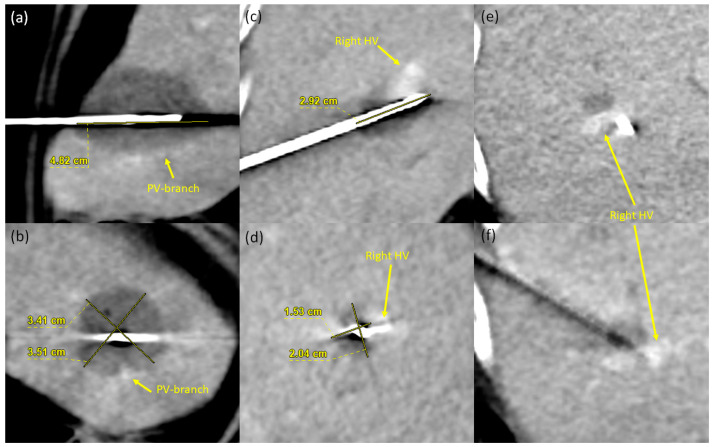
Two example cases showing the large range of AZVs. Both ablations were performed in the ‘standard mode’ with 65 W for 10 min. (**a**,**b**) Ablations of an HCC in a 72-year-old female, with an ablation size of 4.8 × 3.5 × 3.4 cm^3^ (AZV 29.9 mL). At the caudal edge of the ablation site, it is imprinted by a portal vein branch. (**c**–**f**) Ablation of a perivascular residual pancreatic cancer metastasis after previous neoadjuvant chemotherapy in a 73-year-old female with an AZ of 2.9 × 2 × 1.5 cm^3^ (AZV 4.6 mL). Note the placement of the ablation probe immediately adjacent to the right hepatic vein.

A large range in AZ sizes was noted (Figure 2): The mean AZV achieved was 14.3 ± 8.5 mL (range 3.2–49.2 mL). The AZ length was 3.6 ± 0.6 cm (range 2.4–5.6 cm), the long width was 2.7 ± 0.5 cm (range 1.7–4.3), and the short width was 2.5 ± 0.5 cm (range 1.4–3.9 cm).

**Figure 3:**
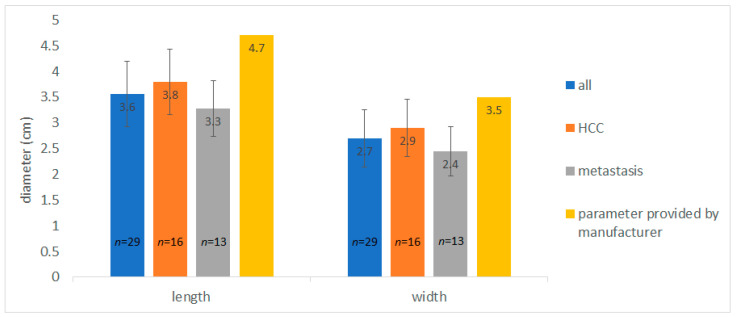
With ablation at 65 W for 10 min, the AZ sizes achieved were smaller than those specified by the manufacturer.

Ablation performed in the ‘standard mode’ (65 W for 10 min) achieved an AZV of 13.9 ± 8.8 mL (*n* = 29), with 16.9 ± 10.1 mL for HCC (*n* = 16) and 10.1 ± 5.1 mL for liver metastasis (*n* = 13). The corresponding AZ length was 3.6 ± 0.6 cm, 3.8 ± 0.6 cm for HCC, and 3.3 ± 0.5 cm for liver metastases. The maximum AZ width was 2.7 ± 0.6 cm overall, 2.9 ± 0.6 cm for HCC, and 2.4 ± 0.5 cm for liver metastases. Thus, both the overall AZ length and width measured in this study were 23% below the values stated by the manufacturer (4.7 cm for length, 3.5 cm for width; Figure 3).

Ablation in the ‘surgical mode’ (95 W for 1 min, followed by 65 W for 10 min) was conducted in nine cases and achieved only a slightly higher AZV of 15.6 ± 7.8 mL (*p* = 0.6), mainly due to a slightly higher AZ length (Table 2). Even when considered separately for HCC (22.5 ± 8.3 mL vs. 16.9 ± 10.1 mL, *p* = 0.38) and liver metastases (12.2 ± 5.2 mL vs. 10.1 ± 5.1 mL, *p* = 0.43), the differences were not significant (*t* tests).

The AZ border distal to the needle tip exceeded 0.6 cm in 4/32 ablations (2 of them in the ‘surgical mode’), which corroborates with the manufacturer’s statement concerning the PR probe. The mean value was only slightly higher for ablation in the ‘surgical mode’ than in the ‘standard mode’ (0.6 ± 0.3 cm vs. 0.5 ± 0.3 cm).

AZVs were significantly larger for ablation of HCCs than for ablation of liver metastases (17.8 ± 9.9 mL vs. 10.1 ± 5.1 mL, *p* = 0.01, *t*-test, Table 2), and they were significantly smaller with a perivascular tumour location (11.7 ± 6.1 mL vs. 18.7 ± 10.4 mL, *p* = 0.01, *t*-test, Table 2). A heat-sink effect, defined as a vessel still perfused and protruding into the AZ, was detected in 20/24 ablations of perivascular lesions and in 0 non-perivascular lesions. Thus, the groups perivascular tumour localisation and heat-sink effect were mostly identical. In the presence of a heat-sink effect, the AZs were significantly smaller, especially in the shorter AZ width (2.3 ± 0.5 vs. 2.8 ± 0.4 cm, *p* = 0.004, Table 2). The heat-sink effect occurred on average at a distance of 0.8 ± 0.3 cm (range: 0.4–1.4 cm) from the emitting point of the needle. In the perivascular group, AZ sizes did not differ significantly between lesions adjacent to the portal vein or the hepatic vein (AZV: 12.5 ± 6.0 mL vs. 11.2 ± 6.3 mL, *p* = 0.62; AZ length: 3.4 ± 0.4 vs. 3.4 ± 0.6 cm, *p* = 0.98; AZ long width: 2.7 ± 0.4 cm vs. 2.5 ± 0.6 cm, *p* = 0.42; AZ short width: 2.5 ± 0.4 cm vs. 2.3 ± 0.5 cm, *p* = 0.35).

Subcapsular tumour localisation was present in 7/14 non-perivascular tumours and 9/24 perivascular tumours. In the presence of subcapsular tumour localisation, the AZVs were slightly larger but not significantly (16.6 mL ± 9.5 mL vs. 12.3 ± 7.5 mL, *p* = 0.15).

The sphericity index was 0.52 ± 0.12 and did not differ significantly between the different groups (Table 2).

Thus, the main factors influencing AZV appear to be tumour type (HCC vs. metastasis) and tumour location (perivascular vs. non-perivascular), which is why the respective subgroups were examined here (Table 3). For further evaluation, a two-way ANOVA was performed, including these two independent variables. The overall model was significant for AZV and AZ length and width (ablation volume: F = 4.6, *p* = 0.009, corrected R^2^ = 0.225, *n* = 38). For AZV, there were significant differences between HCC vs. metastatic ablation (F = 6.0, *p* = 0.02, η_p_^2^ = 0.15) and between perivascular vs. non-perivascular tumour location (F = 4.4, *p* = 0.044, η_p_^2^ = 0.11). No significant interaction was found between the tumour type and tumour location on the AZ size (F = 0.6, *p* = 0.44, η_p_^2^ = 0.018). The effect size f according to Cohen is 0.42 for HCC vs. metastasis, which corresponds to a strong effect according to Cohen’s classification. For perivascular vs. non-perivascular, it is 0.36, which corresponds to a medium effect.

At least a moderate steatosis (CT number in non-contrast-enhanced CT ≤ 40 HU, [18]) was present in five cases. In our study, no significant correlation was found between the CT number in the unenhanced CT and AZV (Pearson correlation: −0.122, *p* = 0.47).

Complications, which were possibly attributed to microwave ablation, occurred in four patients. In one case, a mild hemiparesis with hemihypaesthesia on the left side occurred in the presence of a PFO, which completely regressed after the procedure (grade 3 according to the CIRSE Classification System for Complications, [19]). The AZ was adjacent to the hepatic vein. In two other cases, febrile infections transpired post-intervention, which could be successfully treated with antibiotics, in one of them with accompanying mild hydropic decompensation. In another case, a major perfusion defect was found due to periinterventional thrombosis of a portal vein branch running through the AZ, but no further clinical consequences were observed.

## 4. Discussion

In this retrospective study, the AZVs achievable with the NeuWave PR needle were smaller than predicted by the manufacturer’s data (overall both AZ length and AZ width were 23% below the predicted value), which is based on ex vivo bovine experiments and which concurs with the results of other research groups in clinical studies [4,5], but also in in vivo animal studies [20,21]. This is probably due to the ex vivo non-existent liver perfusion with only minimal heat dissipation. This finding is also consistent with the results of Huber et al. [6], who used retrospective clinical data to calculate a normogram for AZV for the NeuWave PR needle. For ablation with 65 W for 10 min, the normogram results in a volume of 13.4 mL, which is similar to the AZV of 13.9 ± 8.8 mL we found. The sphericity index (0.526 ± 0.12) was similar to the results reported by Winokur et al. [5] for the NeuWave PR needle (0.49).

In addition to ablation in the ‘standard mode’ with 65 W for 10 min, ablation in the ‘surgical mode’ (95 W for 1 min, followed by 65 W for 10 min) was also included. By yielding larger AZs, this ‘surgical mode’ could extend the application range of the PR needle while maintaining the advantage of distal energy control. However, there were no significant differences compared to the ‘standard mode’, even when subgroup analyses were performed. The probable rationale for this is the asymptotic progression of AZ sizes towards a maximum with longer ablation durations [15,16], indicating that ablation with only an initially higher power has no significant effect. However, the PR needle is not technically designed for continuous ablation at 95 W.

Remarkably, larger AZs were attained in the ablation of HCCs than in the ablation of metastases. Liver cirrhosis was present in all patients with HCCs but in one patient only with metastases. Because most of the AZV consists of the liver tissue surrounding the tumour (mean 88.5% in our study), its characteristics play a greater role than those of the tumour itself [10,13]. The differences are therefore likely attributed largely to the different properties of cirrhotic liver tissue (blood perfusion, thermal conductivity, and electrical conductivity). A lower perfusion has been demonstrated for cirrhotic liver tissue than for healthy liver tissue [22,23]. In a physical model that considers this fact, a 36% larger AZ was predicted for ablation in liver cirrhosis [10].

Additionally, according to this model, electrical conductivity only played a minor role, whereas reduced thermal conductivity led to an increased AZ size. This is probably primarily due to the outer zone expansion, in which heating no longer occurs via direct heating but via thermal conductivity. This is particularly reduced in steatosis [10,24], although lower conductivity has also been postulated for liver cirrhosis [25]. However, we could not show a significant influence of moderate-to-severe steatosis (HU ≤ 40 in non-contrast CT) on AZ sizes, probably due to the low presence in our sample (five patients).

Another difference between HCCs and metastases is the pseudocapsule often present in HCCs, which is suspected to have reduced thermal conductivity. Based on this, an ‘oven effect’ was described by Livraghi et al. [26], who observed in RFA that the area of necrosis adapted to the tumour size and shape. For RFA, how much heat can actually be retained by a pseudocapsule remains unclear [13]. However, because the MWA in the area of direct heating is not dependent on thermal conductivity and this area usually already exceeds the tumour boundaries [2], this effect probably only plays a subordinate role. Moreover, in our study, it was not observed that AZ adapted to the tumour boundaries.

Previously published clinical data on the influence of liver cirrhosis are inhomogeneous and insufficient. Heerink et al. [13] and Shyn et al. [27] reached a similar conclusion in retrospective studies. Conversely, Amabile et al. [17] showed larger AZs for HCCs with longer ablation times than for liver metastases, but only centrally located tumours were included in this study. Huber et al. [6] also examined ablation performed with the NeuWave PR needle, while Ruiter et al. [7] investigated ablation performed with a not-further-specified NeuWave ablation device. Both found no significant difference. However, the majority of these studies included cases with many different ablation parameters, and the calculation was based on the AZV-to-energy ratio, without taking specific ablation power or time into account. Additionally, in the studies by Heerink et al. [13] and Ruiter et al. [7], many tumours were treated with multiple needle positions with AZ measurements after the complete ablation.

Another noteworthy finding from the study was a significantly lower AZV observed in cases of perivascular tumour localisation. This outcome was evident in both the *t*-test and two-way ANOVA when considering the parameters of tumour type and perivascular tumour localisation. Thus, this parameter seems to have an influence on the area size that is independent of tumour type. In two cases of perivascular tumour localisation, there was no heat-sink effect (defined as a still-open vessel within the AZ or as an indentation of the AZ by a vessel). Otherwise, both groups were identical, and thus the results for the AZ sizes were similar. Interestingly, perivascular tumour location did not only decrease the short width but also the length and long width of the AZ. Therefore, macrovessel proximity appears to lead to an increased heat removal, which then manifests in a smaller AZ. Poch et al. [9] demonstrated a cooling effect in vivo in pigs due to vessels in the zone of incomplete cell death, but not in the zone of complete cell death. A cooling effect was also predicted in physical models of Deshavar et al. [10] and Tucci et al. [11]. Additionally, Cafarchio et al. [12] predicted an effect of the angle between ablation needle and blood flow direction, which we did not evaluate in our study. However, one would assume different angles when ablating lesions near the portal vein vs. ablating near the hepatic vein due to the liver anatomy. The ablation sizes between these groups did not differ, so our, in that respect limited, data does not support this prediction. To the best of our knowledge, no clinical study has explored the influence of perivascular tumour localisation on the AZ size yet. However, Urbonas et al. [28] described a higher local recurrence rate for tumours adjacent to hepatic veins, whereas Shady et al. [29] found no effect of perivascular tumour localisation on the local recurrence rate.

Subcapsular tumour localisation tended to demonstrate larger AZs, albeit not significantly. This is probably because these tumours were less frequently located perivascularly and more frequently HCCs; thus, a cross correlation was probable.

Based on our results for the shortest diameter of the AZ (short width) and considering a safety margin of 5 mm, HCC in non-perivascular localisation with a maximum size of 2 cm can be treated with a single-needle ablation without re-ablation using the PR needle. In perivascular tumour localisation, this is possible up to a maximum size of 1.6 cm. However, adequate treatment of metastases (safety margin 10 mm) is barely achievable with a single ablation (maximum tumour size: non-perivascular 0.6 cm and perivascular 0.2 cm).

We maintained an acceptable complication rate (four cases, 11%), none of which had permanent sequelae. The complication rate was in line or lower compared with other studies with rates ranging from 3.2% [30] to 10.2% [31] to 37% [29].

The first limitation of this study is its retrospective nature, indicating that possible cofactors could not be adequately controlled. However, a prospective investigation of the influencing factors, particularly tumour localisation, is ethically difficult. Another important limitation of this study is that tissue shrinkage was not considered when measuring AZ sizes. In an in vivo study in pigs, a volume reduction of the ablated area of up to 12% was reported [32]; in a clinical retrospective study, a contraction of the AZ of 7% was found [33]. Accordingly, the AZ sizes that were measured probably need to be corrected slightly upwards. Additionally, AZVs were calculated based on the assumption of an ellipsoid ablation zone and using only the parameters length, long width, and short width. However, the real AZV might differ from this value; in particular, a localized heat-sink effect might cause a deviation. The angle between ablation needle and blood flow direction in perivascular lesions, which is also a possible influencing factor, was not evaluated in this study. One advantage of this study is that only ablation with two specific, similar ablation parameters and only one needle was used, which reduces the corresponding confounding factors in the analysis. On the other hand, it is conceivable that the effects observed here may be different for other ablation parameters and needle types. Moreover, a higher number of cases would certainly be desirable to allow a more differentiated analysis of subgroups.

## 5. Conclusions

In conclusion, the AZs for the NeuWave PR needle were smaller (23% for both AZ length and width) than those predicted by the manufacturer. The distal energy control feature, that is, an extension of the AZ distal to the needle tip of a maximum of 0.5 cm, worked well. The use of the ‘surgical mode’ did not result in a significant enlargement of the AZ. A significantly larger AZ was achieved for ablation of HCCs than for the ablation of metastases. Another significant influencing factor was perivascular tumour localisation, which led to smaller AZ sizes. Ultimately, however, further and larger studies are warranted to further investigate these influencing factors, as well as with other ablation parameters/needles.

## Figures and Tables

**Table 1:** Baseline characteristics.

Baseline Characteristics		HCC (*n* = 19)	Metastasis (*n* = 19)	*p* Value
Age (years)		70.2 ± 9.0	64.2 ± 9.5	0.06
Tumour size (cm)		1.72 ± 0.39	1.21 ± 0.50	0.001
Sex (male)		13 (68.4%)	11 (57.9%)	0.737
Tumour type	HCC	19 (100%)		
	CCC		1 (5.3%)	
	CRC		7 (36.8%)	
	PDAC		6 (31.6%)	
	PNET		2 (10.5%)	
	GNET		1 (5.3%)	
	UC		1 (5.3%)	
	NSCLC		1 (5.3%)	
Hypervascular tumour		19 (100%)	6 (31.6%)	<0.001
Previous local therapy for other lesions		12 (63.2%)	11 (57.9%)	1
Previous systemic therapy		1 (5.3%)	17 (89.5%)	<0.001
Cirrhosis		19 (100%)	1 (5.3%)	<0.001
Steatosis		3 (15.8%)	2 (10.5%)	1
Portal vein hypertension		12 (63.2%)	0 (0%)	<0.001
Perivascular location		10 (52.6%)	14 (73.7%)	0.313
Pericapsular location		11 (57.9%)	5 (26.3%)	0.078
Ablation in the ‘surgical mode’		3 (15.8%)	6 (31.6%)	0.447

Continuous variables are displayed as means ± standard deviation. For continuous variables, *t*-test was performed, and for categorical variables, Yates chi-squared test was conducted. HCC: hepatocellular carcinoma; CCC: cholangiocarcinoma; CRC: colorectal carcinoma; PDAC: pancreatic ductal adenocarcinoma; PNET: pancreatic neuroendocrine tumour; GNET: gastric neuroendocrine tumour, UC: urothelial carcinoma; NSCLC: non-small-cell lung cancer.

**Table 2:** Overall AZ sizes and divided into subgroups. The groups were compared using the *t*-test. Significance was assumed for *p* < 0.05, which is highlighted.

Groups	Volume (mL)	Length (cm)	Long Width (cm)	Short Width (cm)	Sphericity Index
Total (*n* = 38)	14.3 ± 8.5	3.6 ± 0.6	2.7 ± 0.5	2.5 ± 0.5	0.52 ± 0.12
‘Standard mode’ (*n* = 29)	13.9 ± 8.8	3.6 ± 0.6	2.7 ± 0.6	2.5 ± 0.5	0.53 ± 0.12
‘Surgical mode’ (*n* = 9)	15.6 ± 7.8	3.9 ± 0.5	2.7 ± 0.5	2.6 ± 0.5	0.47 ± 0.13
*p* value	0.60	0.13	0.82	0.59	0.19
HCC (*n* = 19)	17.8 ± 9.9	3.9 ± 0.6	2.9 ± 0.5	2.7 ± 0.5	0.54 ± 0.11
Metastasis (*n* = 19)	10.1 ± 5.1	3.4 ± 0.5	2.5 ± 0.5	2.3 ± 0.4	0.5 ± 0.13
*p* value	0.01	0.017	0.007	0.006	0.25
Perivascular (*n* = 24)	11.7 ± 6.1	3.4 ± 0.5	2.6 ± 0.5	2.3 ± 0.5	0.51 ± 0.14
Non-perivascular (*n* = 14)	18.7 ± 10.4	4.0 ± 0.7	2.9 ± 0.5	2.8 ± 0.4	0.53 ± 0.10
*p* value	0.012	0.005	0.042	0.003	0.68
Heat-sink-effect (*n* = 20)	11.6 ± 6.4	3.4 ± 0.5	2.6 ± 0.6	2.3 ± 0.5	0.50 ± 0.15
No heat-sink-effect (*n* = 18)	17.3 ± 9.6	3.9 ± 0.7	2.9 ± 0.5	2.8 ± 0.4	0.54 ± 0.09
*p* value	0.037	0.033	0.069	0.004	0.32
Subcapsular (*n* = 16)	16.6 ± 9.5	3.8 ± 0.7	2.9 ± 0.4	2.7 ± 0.4	0.55 ± 0.11
Non-subcapsular (*n* = 22)	12.3 ± 7.5	3.5 ± 0.6	2.6 ± 0.6	2.4 ± 0.6	0.50 ± 0.13
*p* value	0.15	0.19	0.08	0.06	0.27

**Table 3:** AZ sizes in the different subgroups according to the tumour type and tumour location.

AZ Size	Groups	HCC	Metastasis
*n*	Non-perivascular	9	5
	Perivascular	10	14
Volume (mL)	Non-perivascular	21.7 ± 11.9	13.3 ± 2.7
	Perivascular	14.2 ± 6.2	9.9 ± 5.5
Length (cm)	Non-perivascular	4.2 ± 0.7	3.6 ± 0.4
	Perivascular	3.6 ± 0.4	3.3 ± 0.5
Long Width (cm)	Non-perivascular	3.1 ± 0.5	2.6 ± 0.1
	Perivascular	2.8 ± 0.5	2.4 ± 0.5
Short Width (cm)	Non-perivascular	3.0 ± 0.5	2.6 ± 0.1
	Perivascular	2.6 ± 0.5	2.2 ± 0.5

Means ± standard deviation.

## Data Availability

Data are contained within the article.

## References

[1] Facciorusso A., Di Maso M., Muscatiello N. (2016). Microwave Ablation versus Radiofrequency Ablation for the Treatment of Hepatocellular Carcinoma: A Systematic Review and Meta-Analysis. Int. J. Hyperth..

[2] Kim C. (2018). Understanding the Nuances of Microwave Ablation for More Accurate Post-Treatment Assessment. Future Oncol..

[3] Ryan M.J., Willatt J., Majdalany B.S., Kielar A.Z., Chong S., Ruma J.A., Pandya A. (2016). Ablation Techniques for Primary and Metastatic Liver Tumors. World J. Hepatol..

[4] Ruiter S.J.S., Heerink W.J., de Jong K.P. (2019). Liver Microwave Ablation: A Systematic Review of Various FDA-Approved Systems. Eur. Radiol..

[5] Winokur R.S., Du J.Y., Pua B.B., Talenfeld A.D., Sista A.K., Schiffman M.A., Trost D.W., Madoff D.C. (2014). Characterization of in Vivo Ablation Zones Following Percutaneous Microwave Ablation of the Liver with Two Commercially Available Devices: Are Manufacturer Published Reference Values Useful?. J. Vasc. Interv. Radiol..

[6] Huber T.C., Miller G., Patrie J., Angle J.F. (2021). Relationship of Antenna Work and Ablation Cavity Volume Following Percutaneous Microwave Ablation of Hepatic Tumors. J. Vasc. Interv. Radiol..

[7] Ruiter S.J.S., De Jong J.E., Pennings J.P., De Haas R.J., De Jong K.P., Ruiter S.J.S., De Jong J.E., Pennings J.P., De Haas R.J., De Jong K.P. (2022). Comparison of Two 2.45 GHz Microwave Ablation Devices with Respect to Ablation Zone Volume in Relation to Applied Energy in Patients with Malignant Liver Tumours. Cancers.

[8] Paolucci I., Ruiter S.J.S., Freedman J., Candinas D., de Jong K.P., Weber S., Tinguely P. (2022). Volumetric Analyses of Ablation Dimensions in Microwave Ablation for Colorectal Liver Metastases. Int. J. Hyperth..

[9] Poch F.G.M., Geyer B., Neizert C.A., Gemeinhardt O., Niehues S.M., Vahldiek J.L., Frericks B., Lehmann K.S. (2021). Periportal Fields Cause Stronger Cooling Effects than Veins in Hepatic Microwave Ablation: An in Vivo Porcine Study. Acta Radiol..

[10] Deshazer G., Merck D., Hagmann M., Dupuy D.E., Prakash P. (2016). Physical Modeling of Microwave Ablation Zone Clinical Margin Variance. Med. Phys..

[11] Tucci C., Trujillo M., Berjano E., Iasiello M., Andreozzi A., Vanoli G.P. (2022). Mathematical Modeling of Microwave Liver Ablation with a Variable-Porosity Medium Approach. Comput. Methods Programs Biomed..

[12] Cafarchio A., Iasiello M., Vanoli G.P., Andreozzi A. (2023). Microwave Ablation Modeling with AMICA Antenna: Validation by Means a Numerical Analysis. Comput. Biol. Med..

[13] Heerink W.J., Solouki A.M., Vliegenthart R., Ruiter S.J.S., Sieders E., Oudkerk M., de Jong K.P. (2018). The Relationship between Applied Energy and Ablation Zone Volume in Patients with Hepatocellular Carcinoma and Colorectal Liver Metastasis. Eur. Radiol..

[14] Singh S., Repaka R., Al-Jumaily A. (2019). Sensitivity Analysis of Critical Parameters Affecting the Efficacy of Microwave Ablation Using Taguchi Method. Int. J. RF Microw. Comput.-Aided Eng..

[15] Hines-Peralta A.U., Pirani N., Clegg P., Cronin N., Ryan T.P., Liu Z., Goldberg S.N. (2006). Microwave Ablation: Results with a 2.45-GHz Applicator in Ex Vivo Bovine and in Vivo Porcine Liver. Radiology.

[16] Bedoya M., Del Rio A.M., Chiang J., Brace C.L. (2014). Microwave Ablation Energy Delivery: Influence of Power Pulsing on Ablation Results in an Ex Vivo and in Vivo Liver Model. Med. Phys..

[17] Amabile C., Ahmed M., Solbiati L., Meloni M.F., Solbiati M., Cassarino S., Tosoratti N., Nissenbaum Y., Ierace T., Goldberg S.N. (2017). Microwave Ablation of Primary and Secondary Liver Tumours: Ex Vivo, in Vivo, and Clinical Characterisation. Int. J. Hyperth..

[18] Boyce C.J., Pickhardt P.J., Kim D.H., Taylor A.J., Winter T.C., Bruce R.J., Lindstrom M.J., Hinshaw J.L. (2010). Hepatic Steatosis (Fatty Liver Disease) in Asymptomatic Adults Identified by Unenhanced Low-Dose CT. Am. J. Roentgenol..

[19] Filippiadis D.K., Binkert C., Pellerin O., Hoffmann R.T., Krajina A., Pereira P.L. (2017). Cirse Quality Assurance Document and Standards for Classification of Complications: The Cirse Classification System. Cardiovasc. Interv. Radiol..

[20] Hui T.C.H., Brace C.L., Hinshaw J.L., Quek L.H.H., Huang I.K.H., Kwan J., Lim G.H.T., Lee F.T., Pua U. (2020). Microwave Ablation of the Liver in a Live Porcine Model: The Impact of Power, Time and Total Energy on Ablation Zone Size and Shape. Int. J. Hyperth..

[21] Harari C.M., Magagna M., Bedoya M., Lee F.T., Lubner M.G., Louis Hinshaw J., Ziemlewicz T., Brace C.L. (2016). Microwave Ablation: Comparison of Simultaneous and Sequential Activation of Multiple Antennas in Liver Model Systems. Radiology.

[22] Van Beers B.E., Leconte I., Materne R., Smith A.M., Jamart J., Horsmans Y. (2001). Hepatic Perfusion Parameters in Chronic Liver Disease. Am. J. Roentgenol..

[23] Hashimoto K., Murakami T., Dono K., Hori M., Kim T., Kudo M., Marubashi S., Miyamoto A., Takeda Y., Nagano H. (2006). Assessment of the Severity of Liver Disease and Fibrotic Change: The Usefulness of Hepatic CT Perfusion Imaging. Oncol. Rep..

[24] Ahmed M., Liu Z., Humphries S., Goldberg S.N. (2008). Computer Modeling of the Combined Effects of Perfusion, Electrical Conductivity, and Thermal Conductivity on Tissue Heating Patterns in Radiofrequency Tumor Ablation. Int. J. Hyperth..

[25] Liu Z., Ahmed M., Weinstein Y., Yi M., Mahajan R.L., Goldberg N.S. (2006). Characterization of the RF Ablation-Induced “Oven Effect”: The Importance of Background Tissue Thermal Conductivity on Tissue Heating. Int. J. Hyperth..

[26] Livraghi T., Goldberg S.N., Lazzaroni S., Meloni F., Solbiati L., Gazelle G.S. (1999). Small Hepatocellular Carcinoma: Treatment with Radio-Frequency Ablation versus Ethanol Injection. Radiology.

[27] Shyn P.B., Bird J.R., Koch R.M., Tatli S., Levesque V.M., Catalano P.J., Silverman S.G. (2016). Hepatic Microwave Ablation Zone Size: Correlation with Total Energy, Net Energy, and Manufacturer-Provided Chart Predictions. J. Vasc. Interv. Radiol..

[28] Urbonas T., Anderson E.M., Gordon-Weeks A.N., Kabir S.I., Soonawalla Z., Silva M.A., Gleeson F.V., Reddy S. (2019). Factors Predicting Ablation Site Recurrence Following Percutaneous Microwave Ablation of Colorectal Hepatic Metastases. HPB.

[29] Shady W., Petre E.N., Do K.G., Gonen M., Yarmohammadi H., Brown K.T., Kemeny N.E., D’Angelica M., Kingham P.T., Solomon S.B. (2018). Percutaneous Microwave versus Radiofrequency Ablation of Colorectal Liver Metastases: Ablation with Clear Margins (A0) Provides the Best Local Tumor Control. J. Vasc. Interv. Radiol..

[30] Dou J.-P., Yu J., Yang X.-H., Cheng Z.-G., Han Z.-Y., Liu F.-Y., Yu X.-L., Liang P. (2017). Outcomes of Microwave Ablation for Hepatocellular Carcinoma Adjacent to Large Vessels: A Propensity Score Analysis. Oncotarget.

[31] Livraghi T., Meloni F., Solbiati L., Zanus G. (2012). Complications of Microwave Ablation for Liver Tumors: Results of a Multicenter Study. Cardiovasc. Interv. Radiol..

[32] Erxleben C., Niehues S.M., Geyer B., Poch F., Bressem K.K., Lehmann K.S., Vahldiek J.L. (2021). CT-Based Quantification of Short-Term Tissue Shrinkage Following Hepatic Microwave Ablation in an in Vivo Porcine Liver Model. Acta Radiol..

[33] Lee J.K., Siripongsakun S., Bahrami S., Raman S.S., Sayre J., Lu D.S. (2016). Microwave Ablation of Liver Tumors: Degree of Tissue Contraction as Compared to RF Ablation. Abdom. Radiol..

